# Fiber photometry-based investigation of brain function and dysfunction

**DOI:** 10.1117/1.NPh.11.S1.S11502

**Published:** 2023-11-14

**Authors:** Nicole Byron, Shuzo Sakata

**Affiliations:** University of Strathclyde, Strathclyde Institute of Pharmacy and Biomedical Sciences, Glasgow, United Kingdom

**Keywords:** fiber photometry, calcium imaging, cell type, astrocyte, neural oscillation

## Abstract

Fiber photometry is an optical method to monitor fluorescent signals using a fiber optic cannula. Over the past two decades, together with the development of various genetically encoded biosensors, it has been applied to investigate various types of activity in the central nervous system. This includes not only type-specific neuronal population activity, but also non-neuronal activity and neurotransmitter/neuropeptide signals in awake, freely behaving animals. In this perspective, we summarize the recent development of this technique. After describing common technical pitfalls, we discuss future directions of this powerful approach for investigating brain function and dysfunction.

## 1 Introduction

The development of neural interface technologies has been an active research area.^1^^,^^2^ Historically, this field can be dated back to Luigi Galvani’s experiments in the 18^th^ century. While electrophysiology continues to be the gold standard for monitoring individual neuronal activity in living brain tissue at high temporal resolution, optical approaches have unique advantages over electrophysiology. Optical monitoring and manipulation of neuronal activity have been routinely performed in a cell-type-specific fashion by expressing genetically encoded actuators and sensors.^3^^–^^8^ Out of various optical approaches, fiber photometry offers a simple, but powerful solution to monitor type-specific neuronal population activity in freely behaving animals.

**Fig. 1:**
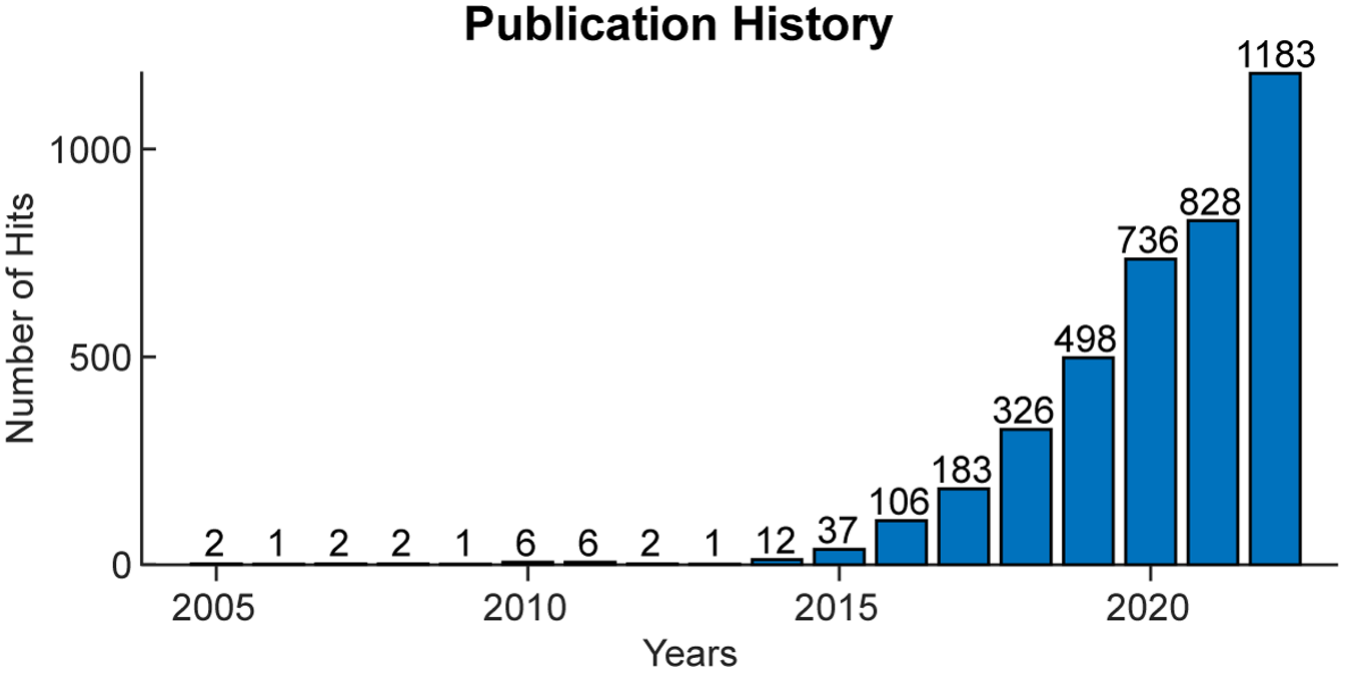
Increased interest in fiber photometry over recent years. Total number of hits from PubMed, Web of Science, and Google Scholar when searching either “fiber photometry” or “fibre photometry” across all available years.

Fiber photometry was first introduced to neuroscience in 2005.^9^ The advent of genetically encoded calcium indicators (GECIs) allows fiber photometry to monitor cell type-specific population activity from deep brain regions in freely behaving mice.^10^^,^^11^ Fiber photometry has been widely adopted to characterize neural population activity in behaving animals along with opto- and chemo-genetic experiments over the past two decades (Fig. 1).

**Fig. 2:**
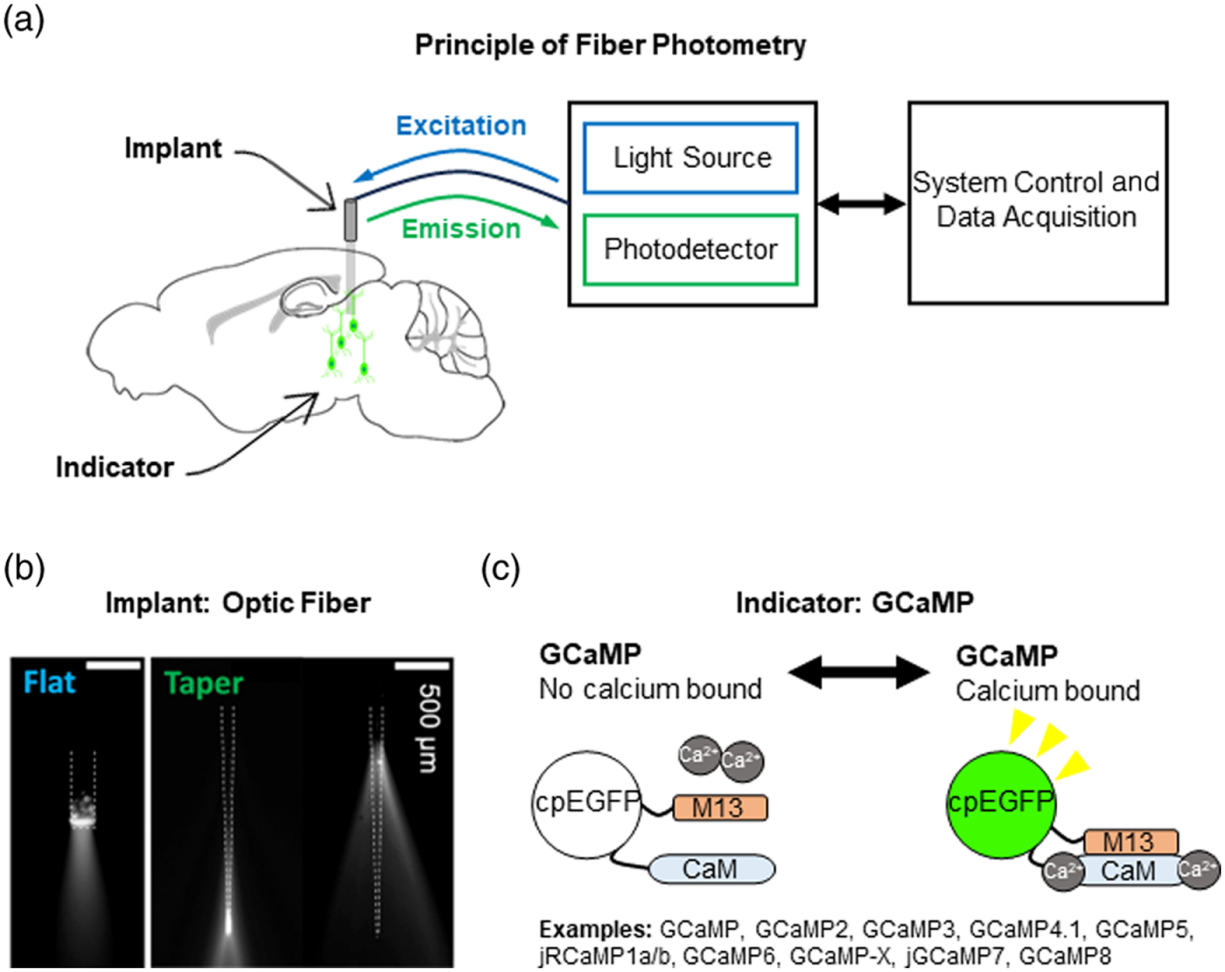
General principle of fiber photometry in neuroscience. (a) Principle of fiber photometry. Light from a light source is passed through a fiber optic implant to excite fluorescent indicators. Emitted light passes back through the fiber optic implant and is collected at a photodetector. Signals will be transferred to a computer for data analysis. All systems will be varied and include other optical elements. (b) Commonly used flat multimode fiber (left) and newly developed and established tapered optic fiber^12^ (right). Images of the tapered optic fibers light profile illustrate the depth resolution capacity. (c) GCaMP fluorescence occurs from a fluorescence protein (circularly permuted enhanced green fluorescent protein, cpEGFP) after calcium binds.

Fiber photometry typically involves two major components (Fig. 2): fluorescent indicators and optical devices. The former can be either chemical indicators or genetically encoded sensors. While the pioneering study used calcium-sensitive dyes,^9^ GCaMPs are the most popular choice [Fig. 2(c)]. Genetically engineered voltage indicators have also been deployed to monitor fast neural oscillations.^13^^,^^14^ Over the past 5 years, the use of genetically encoded sensors for neurotransmitters and neuromodulators has gained popularity.^5^^,^^15^

In typical photometry experiments, fiber optics delivers and collects photons in brain tissue [Fig. 2(a)]. While conventional multimode fibers are commonly used, a tapered fiber allows depth-resolved light delivery and collection in freely behaving mice, with reduced tissue damage [Fig. 2(b)].^12^^,^^16^ Applications of micro-photodiodes are also on the horizon.^17^^,^^18^ Thus, by combining genetically encoded sensors and advanced optical technologies, fiber photometry is a highly versatile approach to investigate neural functions.

In this perspective, we summarize two emerging applications: fiber photometry in non-neuronal cells and multi-modal monitoring of neural activity. After commenting on potential technical pitfalls, we will discuss future directions of fiber photometry.

## 2 Fiber Photometry-Based Investigation of Non-Neuronal Cell Activity

The nervous system consists of not only neurons, but also glial cells. Astrocytes are the most abundant glial cells in the brain and play diverse roles, including cognitive functions.^19^^–^^21^ Despite the importance of astrocytes, monitoring astrocyte activity was impractical for many years as they are not electrically excitable, meaning common electrophysiological methods were not applicable. However, their ability to exhibit rich intracellular calcium signals means they can be interrogated using new optical tools that allow for fluorescent indicators to be expressed by astrocytes. Thus, GECIs have been applied to study astrocyte function *in vivo*.^22^

Recently, Tsunematsu and her colleagues^23^ utilized YC-nano50 to investigate region-specific and state-dependent calcium dynamics in astrocytes across sleep-wake cycles. YC-nano50 is a different category of GECIs since two fluorescent proteins are encoded and fluorescent signals are emitted based on Förster resonance energy transfer (FRET).^24^^,^^25^ Thus, YC-nano50 allows ratiometric photometry by monitoring two fluorescent signals with a single excitation wavelength.

By expressing YC-nano50 across the brain by crossing two transgenic mouse lines, Tsunematsu and her colleagues^23^ monitored calcium signals across multiple brain regions and sleep-wake cycles. Previously, astrocyte calcium concentration was thought to increase during wakefulness and decrease during sleep.^26^ However, they discovered that although cortical and cerebellar astrocytes exhibit state-dependent calcium signals as predicted, astrocytes in the hypothalamus and pons exhibited distinct patterns: calcium signals remain high even during non-rapid eye movement sleep whereas they decrease during rapid eye movement (REM) sleep.

Although this study demonstrated region-specific and state-dependent astrocytic calcium dynamics for the first time, there are at least three limitations in this study. First, pH influences the fluorescence properties of fluorophores differently and pH can change throughout the sleep-wake cycle.^27^^,^^28^ Ratiometric measurements of fluorescent signals across different vigilance states should be interpreted carefully. Indeed, although several experimental variables (e.g., stereotaxic coordinates and astrocyte classes) are not the same, using GCaMPs, a recent study observed state-dependent calcium dynamics in the pons with higher calcium signals in both wakefulness and REM sleep.^29^ This discrepancy must be resolved in the near future by monitoring astrocyte calcium signals across multiple brainstem nuclei. It may be worth referencing GFP signals to evaluate factors independent of calcium signals. Second, since astrocytes are diverse cell populations,^30^^–^^32^ the findings cannot be generalized. It is imperative to characterize calcium signals across different astrocyte types. Finally, photometry provides calcium signals only from cell populations. Because astrocyte calcium signals differ depending on cellular locations,^33^^,^^34^ monitoring calcium signals by optical means with high spatial resolution is required in the future. Despite these limitations and challenges, detailed characterization of astrocyte calcium dynamics across multiple brain regions and astrocyte types will lead to a better understanding of brain functions in health and disease.

## 3 Simultaneous *In Vivo* Electrophysiology and Fiber Photometry

A major drawback of GCaMP-based optical monitoring is its low temporal resolution. Genetically encoded voltage indicators have been combined with fiber photometry,^13^^,^^14^ but their adoption is difficult in a conventional laboratory setting. Therefore, combining electrophysiology can be an alternative.

Patel and her colleagues took a simple approach to utilize an “optrode” (a hybrid of an optical fiber and wire electrode) to complement each other. Here, an electrode monitored fast field potentials while an optic fiber allowed cell-type-specific photometry.^35^ As an application, they focused on pontine waves (P-waves). P-waves or ponto-geniculo-occipital waves are ∼100-ms long brain waves and a major electrophysiological marker of REM sleep along with theta oscillations. Despite the prominence of P-waves, their existence in mice was anecdotal for decades. Leveraging their own discovery of P-waves in mice,^36^ they examined if mesopontine cholinergic neurons are involved in mouse P-waves as implicated in other species. Indeed, they found that cholinergic transients are associated with P-waves.^35^^,^^37^

While this particular study was a simple combination of conventional electrophysiology and fiber photometry, other recent studies have monitored spiking activity.^38^^,^^39^ This type of combination of electrophysiology and photometry creates an opportunity where either approach alone cannot answer a particular scientific question.

More specifically, although electrophysiology allows monitoring neuronal signals at microsecond resolution, cell type classification is typically based on spike waveforms. Thus, it is challenging or impractical to extract cell type-specific activity. On the other hand, although GCaMP-based photometry allows monitoring of cholinergic activity, it cannot detect sub-second neural events like P-waves or other neural oscillations, which can be easily detected electrophysiologically. Thus, the combination of electrophysiological and photometry complements each other and creates the opportunity to investigate cell type-specific activity in the context of a certain brain state.

## 4 Technical Considerations

Although fiber photometry is easy to implement in many laboratories, non-specific signals must be taken into consideration to accurately monitor fluorescent signals. Non-specific signals may originate from two sources: movement artifacts and autofluorescence (AF). Since a fiber optic cannula is rigid and fixed to the skull, the relative displacement between the brain tissue and fiber tip results in artificial fluorescent changes. Additionally, the fiber optic is susceptible to subtle bending, which can alter the light output. This can cause artifacts within signal collection. Therefore, it is essential to correct this. Practically, two options can be considered: first, an FRET-based sensor can be employed.^23^^,^^25^ The second and commonly used approach is to use isosbestic illumination, which provides signals independent of indicators.^35^^,^^40^ If such control signals are not measured, careful data interpretation is essential.

The second major source of non-specific signals is AF. The source of AF can be from brain tissue, fiber optics and patch cables.^41^ While a low-AF patch cable is commercially available, regular photobleaching of a patch cable before recording is recommended. Additionally, since the emission spectra of brain tissue AF overlaps with GCaMPs emission spectra,^42^^,^^43^ background fluorescence intensity also decreases as a function of time because of AF photobleaching. Experiments in which several seconds of trials are repeated are less likely to be affected by tissue AF since ΔF/F can be easily obtained. On the other hand, hours-long recordings require not just a high signal-to-noise ratio, but also additional baseline adjustment. Thus, although fiber photometry is a versatile approach, implementation, and signal processing require a basic understanding of the principles of the method.

## 5 Future Directions

While fiber photometry has provided an innovative solution to monitor neurobiological signals in deep brain structures, we expect at least four major domains to be developed further in coming years.

The first are sensors. We expect further development of genetically encoded sensors for a wide variety of biological targets.^5^^,^^15^^,^^44^^–^^47^ In particular, the development of neurotransmitters and neuropeptides has been successful.^5^^,^^15^^,^^48^^–^^52^ These emerging tools have expanded not just to various neurotransmitters and neuropeptides, but also to their excitation/emission spectrum.^50^^,^^52^^,^^53^ These tools now allow us to monitor multiple neurotransmitters simultaneously in behaving animals.^54^^,^^55^ It would be interesting to expand the repertoire to other extracellular molecules, such as cytokines and metabolites. In particular, tracking pathological molecular events in brain disorders, such as Alzheimer’s disease, will provide an opportunity to develop and optimize treatment strategies. In addition to monitoring extracellular molecules, optical monitoring of intracellular signaling is also an exciting avenue. For example, Sabatini and his colleagues^56^ elegantly demonstrated cell type-specific dopamine-induced protein kinase A (PKA) signaling *in vivo*. This approach is based on fluorescence lifetime photometry (FLiP).^57^^,^^58^ Since the fluorescence lifetime of a fluorophore reflects the microenvironment of fluorophores, such as pH changes, ion concentrations, or molecular interactions,^59^^–^^61^ the combination of FLiP with various sensors is a promising direction.

Second, we also expect further development of optical devices. Tapered fibers have already demonstrated depth-resolved photometry in freely behaving animals.^12^ Although the current limitation of this approach is its depth coverage and spatial resolution, it may be interesting to place miniaturized light sources, such as μLED arrays,^62^ along the fiber. Another direction is to adopt microfabrication technologies to integrate both μLED and photodiodes.^17^^,^^18^ Pioneering work has already demonstrated wireless battery-free photometry systems.^18^ Ultimately, microfabrication technologies may allow us to monitor fluorescent signals from individual neurons by deploying single-photon avalanche diode arrays.^17^ In addition, adopting a beam-shaping approach is an attractive possibility.^63^

Third, as we discussed above, combining other modalities, such as electrophysiology, is critical to compensate for the drawback of photometry technology.^35^^,^^38^^,^^64^^,^^65^ In addition to acquiring signals in the brain, controlling neural and non-neuronal signals optically or by other means is also a crucial area to explore.

Finally, since an advantage of photometry is its minimal invasiveness to target deep brain tissue, increasing the scalability is critical to achieving brain-wide functional mapping. Although several pioneering studies have tackled this challenge,^40^^,^^66^^,^^67^ implants and optical devices can be developed further to obtain volumetric signals across the brain. More specifically, because conventional flat fiber-based approaches only allow monitoring activity at the tip of the fiber, depth-resolved photometry is critical.^12^ Scaling up of channels not just in a horizontal plane, but also in a vertical plane is required for brain-wide functional mapping.

In conclusion, since 2005, fiber photometry has been deployed for many experiments. In conjunction with the development of novel genetically encoded sensors and other technologies, fiber photometry will keep playing a significant role in a better understanding of brain function and dysfunction.

## Data Availability

Data sharing is not applicable.
